# Psychological outcomes of patients with bipolar disorder during the perinatal period

**DOI:** 10.1192/j.eurpsy.2025.1044

**Published:** 2025-08-26

**Authors:** S. Arsova, S. Mitrovska, M. Milutinovic, S. Kocoska, V. Isjanovski, S. Bajraktarov, M. Stankovic

**Affiliations:** 1 Day Hospital; 2 Head of University Clinic of Psychiatry, University Clinic of Psychiatry; 3 PHCI Psychiatric Hospital “Skopje”, Skopje, North Macedonia

## Abstract

**Introduction:**

Women with bipolar disorder (BD) face a high risk of adverse mental health outcomes during the perinatal period, including a high prevalence of affective episodes and postpartum psychosis. The challenge of management BD in this period includes balancing maternal stability while securing fetal safety, given the teratogenic risks of mood-stabilizing medications.

**Objectives:**

To evaluate the course of BD during the perinatal period, assess the impact of risk factors, and examine the effectiveness of therapeutic interventions.

**Methods:**

This prospective cohort study included 23 patients with various sociodemographic characteristics, diagnosed with BD (ICD-10 criteria). They were monitored over the course of 6 months utilizing EPDS (Edinburgh Postnatal Depression Scale), and the Young Mania Rating Scale at baseline, 3 – months and 6-months follow up, in addition to a clinically guided interview, non-standardized sociodemographic and risk factors questionnaire. The patients were treated with a combined approach in a specialized unit for mothers, including individual and group therapy, along with pharmacological treatment. Nearly all patients were switched to atypical antipsychotics with stabilizing effects while valproate was excluded due to teratogenic risks.

**Results:**

The cohort comprised mostly of women over 30 (69.6%, p = 0.006), married (78.3%, p = 0.000), with stable socioeconomic status (69.6%, p = 0.008). Combined treatment (inpatient and outpatient) was predominant (82.6%, p = 0.000), with antipsychotics either alone (21.7%) or in combination with other medications as the most common treatment (91.3%, p = 0.000). All participants reported risk factors, including history of affective episodes (95.6%), traumatic events, intimate-partner violence (34.8%), substance abuse (26.1%), and unplanned pregnancy (21.7%).The EPDS scores showed a low likelihood of depression at baseline, 3, and 6 months (56.5%, 69.6%, 69.6%; p < 0.05), with mean scores decreasing from baseline to three months and slightly increasing at six months (p = 0.81). The YMRS scale indicated a significant reduction in mania from baseline to six months (p = 0.038). There was no significant correlation between risk factors and postnatal depression (p > 0.05).

**Conclusions:**

The study confirms that BD women are at high risk for affective destabilization during the perinatal period. While continuous treatment mitigates severe mood deterioration, BD women remain vulnerable, necessitating long-term monitoring. One or more risk factors were registered in all of the participants. Although no direct significant correlation was observed in this study, existing literature suggests that risk factors such as unplanned pregnancy, abrupt medication changes, and intimate-partner violence are associated with adverse outcomes in BD women during the perinatal period. Understanding and managing these risks is essential for comprehensive BD care.

**Disclosure of Interest:**

None Declared

